# Overexpressed HDGF as an independent prognostic factor is involved in poor prognosis in Chinese patients with liver cancer

**DOI:** 10.1186/1746-1596-5-58

**Published:** 2010-09-16

**Authors:** Yanyan Zhou, Nanxiang Zhou, Weiyi Fang, Jirong Huo

**Affiliations:** 1 Department of Gastroenterology, Second Xiangya Hospital of Central South University, People's Road 139, Changsha, 410011, China; 2 Cancer Institute, Southern Medical University, Guangzhou, 510515, China

## Abstract

**Background:**

Hepatoma-derived growth factor (HDGF) is involved in the hepatocarcinogenesis. In this study, we investigated the HDGF expression in hepatocellular carcinoma (HCC) and its correlation with clinicopathologic features, including the survival of patients with HCC. Furthermore, we examined the biological processes regulated by HDGF during the development of using HepG2 cell line as a model system.

**Methods:**

we used immunohistochemistry to compare HDGF protein expression in HCC and normal liver tissues and further analyze the HDGF protein expression in clinicopathologically characterized 137 HCC cases. We stably knocked down the endogenous expression level of HDGF in HepG2 cells with specific shRNA-expressing lentiviral vector. Following the successful establishment of stable cells, we examined *in vitro *cell growth by MTT assay, anchorage-independent growth by soft-agar colony formation assay and cell migration/invasion by transwell and boyden chamber assay. And in addition, we also investigated the *in vivo *tumor growth by xenograft transplantation of HepG2 cells into nude mice.

**Results:**

Protein expression level of HDGF was markedly higher in HCC tissues than that in the normal liver tissues(P = 0.011). In addition, high expression of HDGF protein was positively correlated with T classification(*p *< 0.001), N classification (*p *< 0.001), and clinical stage (*p *< 0.001) of HCC patients. Patients with higher HDGF expression showed a significantly shorter overall survival time than did patients with low HDGF expression. Multivariate analysis suggested that HDGF expression might be an independent prognostic indicator(*p *< 0.001) for the survival of patients with HCC. HDGF-specific shRNA (shHDGF) successfully knocked down its endogenous expression in HepG2 cells. Compared to the parental and control shRNA-transfected (shCtrl) HepG2 cells, the shHDGF cells exhibited significantly reduced *in vitro *cell growth, anchorage-independent growth, cell migration and invasion (*p *< 0.05). *In vivo*, the xenograft transplants from shHDGF cells gave rise to much smaller tumors as compared to those from shCtrl cells.

**Conclusion:**

High HDGF expression is associated with poor overall survival in patients with HCC. Down-regulation of HDGF inhibits the growth, anchorage-independent growth, migration and invasion of HepG2 cells.

## Background

Hepatocellular carcinoma (HCC) is one of the most common malignancies worldwide, especially in Asia [1]. In China, HCC presents with the third highest mortality rate among all malignant carcinomas, following gastric and esophageal cancer, leading to approximately 110,000 deaths every year, which account for 45% of total HCC deaths worldwide [2]. Multiple risk factors have been associated with the initiation and development of HCC, including chronic infection of hepatitis viruses B, C or D, aflatoxin, alcohol abuse, hereditary metabolic liver diseases, diabetes mellitus and etc [1,3]. Similar to most other types of cancer, hepatocarcinogenesis is a multi-step process involving multiple genetic alterations, such as activation of oncogenes and inactivation of tumor suppressor genes, which ultimately lead to malignant transformation of hepatocytes.

Hepatoma-derived growth factor (HDGF) is a heparin-binding protein originally isolated from the conditioned medium of HuH-7 hepatoma cell line [4,5]. Recent studies revealed that HDGF has mitogenic activity for multiple cell types, including HCC cells, fibroblasts, endothelial cells, vascular smooth muscle cells and fetal hepatocytes [4-8]. Besides, HDGF participates in other cellular processes, such as renal development, cardiovascular differentiation, angiogenesis and sensitization of cancer cells to irradiation [9-12]. During cancer development, high levels of HDGF were detected in various human cancers[13], and its level has been demonstrated as a prognostic factor for several cancers including gastric [14], HCC [15], non-small-cell lung cancer [16,17], esophageal carcinoma [18] and pancreatic cancer [19].

In order to clarify the role of HDGF in the pathogenesis of HCC, we investigated the correlation of HDGF protein expression with clinicopathologic features with HCC in Chinese populations. We found that the expression level of HDGF protein was higher in HCC tissues than that in normal liver tissues. High expression of HDGF was associated with poor prognosis of HCC. Moreover, our results indicated that HDGF was involved in progression of HCC by promoting cell growth, invasion and migration of HCC cells. In our investigation, HDGF may act as an oncogene in the pathogenesis of HCC.

## Materials and methods

### Sample collecting

137 paraffin-embedded HCC samples and 49 noncancerous paraffin-embedded normal liver samples were obtained from People's hospital of Hunan Province, China. In 137 HCC cases, there were 87 male and 50 female ranging in age from 14 to 79 years (median, 48 years). For the use of these clinical materials for research purposes, prior consents from the patients and approval from the Ethics Committees of People's hospital of Hunan Province were obtained and all the procedures have been performed in compliance with the Helsinki Declaration. All specimens had confirmed pathological diagnosis and were staged according to the 2002 hepatocellular carcinomas staging system of the International Union Against Cancer(UICC).

### Immunohistochemistry (IHC)

Immunohistochemistry was performed according to according to standard protocol[20-22] Paraffin sections (3 μm) from 137 HCC samples and 49 normal liver samples were deparaffinized in 100% xylene and re-hydrated in descending ethanol series according to standard protocols. Heat-induced antigen retrieval was performed in 10 mM citrate buffer at 100°C for 2 min. Endogenous peroxidase activity and non-specific antigen were blocked with peroxidase blocking reagent containing 3% hydrogen peroxide and serum followed by incubation with rabbit anti-human HDGF antibody (1:100,Proteintech Inc,USA) overnight at 4°C. After washing, the sections incubated with biotin-goat anti-mouse/rabbit antibody at room temperature for 10 minutes were then conjugated with horseradish peroxidase (Maixin Inc, China). The peroxidase reaction was developed with 3, 3-diaminobenzidine chromogen solution in DAB buffer substrate. Sections were visualized with DAB and counterstained with hematoxylin, mounted in neutral gum and analyzed using a bright field microscope.

### Evaluation of staining

The immunohistochemically-stained tissue sections were reviewed and scored separately by two pathologists blinded to the clinical parameters. HDGF expression in the nucleus was independently evaluated. Cases in which <90% and >90% of cancer cells at levels greater than or equal to what is observed in the endothelial cells were regarded as HDGF expression index (EI) levels I and II, respectively[18,19]. HDGF EI was determined separately for the nucleus.

### Cell line

The hepatoma cell line HepG2 was acquired from the Cancer Institute, Central South University and cultured in RPMI1640 medium (HyClone Inc, USA) supplemented with 10% fetal bovine serum (FBS) (*PAA *Laboratories, Inc, Austria) in a 37°C, 5% CO_2 _incubator.

### Extraction of total RNA and reverse transcription followed by PCR (RT-PCR)

Total RNA was extracted from approximately 1 × 10^6 ^cells using Trizol reagent (Invitrogen, USA) following the manufacturer's instructions. cDNA was then synthesized from 2 μmug total RNA using oligo(dT)15 as the primer along with the MMLV reverse transcriptase (Takara Inc, Japan). To determine the steady-state mRNA level of HDGF in HepG2 cells, PCR was performed with the following primers: HDGF forward primer 5'-GAGGGTGACGGTGATAAGAA-3', reverse primer 5'-GAAACATTGGTGGCTACAGG-3', and the amplicon size is 377 bps. GAPDH (internal control) forward primer 5'-TTCGCTCTCTGCTCCTC-3', reverse primer 5'-GATGATCTTGAGGCTGTTGT-3', and the amplicon size is 520 bps. The PCR condition was one cycle of denaturation at 94°C for 2 min, 28 cycles of denaturation at 94°C for 20 seconds (s), annealing at 55°C for 30 s and extension at 72°C for 30 s, followed by one cycle of final extension at 72°C for 10 min. After the PCR reaction, the PCR products was loaded on 1% agarose gel and visualized by ethidium bromide staining.

### Construction of HDGF-shRNA lentiviral vector

The BLOCK-iT RNAi lentiviral expression plasmid was purchased from Invitrogen Inc. The shRNA sequence targeting HDGF (shHDGF) and a control shRNA sequence (shCtrl) targeting no known human genes was designed using the BLOCK iT RNAi Designer http://www.invitrogen.com as follows: shHDGF, sense 5'-CACCGCCGTGAAATCAACAGCCAAAACGTTGGCTGTTGATTTCACGG-3'; antisense 5'-AAAACCGTGAAATCAACAGCCAACTTTTGGCTGTTGATTTCACGGC-3'. shCtrl, sense 5'-CACCGCCCTGAATTGAACAGCCAAAACGTTGGCTGTTGATTTCACGG-3'; 5'-AAAACCGTGAAATCAACAGCCAACTTTTGGCTGTTCAATTCAGGGC-3'. The shRNA primers were cloned into the lentiviral expression plasmid following the manufacturer's instruction and confirmed by sequencing.

### Establishment of HepG2 cell line stably expressing shRNA

shRNA-expressed lentiviral plasmid (either HDGF-specific or control) was transfected into HepG2 cells using Lipofectamin2000 (Invitrogen) according to the Xie's instructions[23]. 48 h later, the cells were subjected to Blasticidin selection at a final concentration of 1.2 μmug/mL. Following two weeks of selection, stable pooled cells were further expanded for future experiments.

### Real-time PCR

Real-time PCR was performed to measure the knock down efficiency of HDGF mRNA expression using SYBR Premix Ex Taq (Takara, Japan) as described previously [22]. The sense primer: 5' CAGCCAACAAATACCAAGTCT 3', Antisense primer: 5' GTTCTCGATCTCCCACAGC 3'. GAPDH gene was used as an inner control. The sense primer: 5' GAAGGTCGGAGTCAACGG 3', Antisense primer: 5' TGGAAGATGGTGATGGGATT 3'.

### Western blot analysis

Approximately 5 × 10^6 ^cells were lysed in RIPA Buffer (50 mM Tris-HCl pH 8.0, 1 mM EDTA pH 8.0, 5 mM DTT, 2% SDS) and total protein concentration determined with BCA assay (Beyotime Inc, China). 60 μmug of total protein were loaded onto 10% SDS-PAGE gel. Antibodies used for Western blot analysis included: rabbit polyclonal anti-HDGF antibody (Proteintech Inc, 1:200), anti-tubulin antibody (Santa Cruz, USA, 1:400), and HRP-conjugated anti-rabbit secondary antibody (Amersham Pharmacia Biotech, 1:50).

### Cell growth analysis

Cell growth was determined by MTT assay (Sigma, USA). Briefly, 1 × 10^3 ^cells were seeded into 96-well plate with quadruplicate for each condition. 72 h later, MTT reagent was added to each well at 5 mg/mL in 20 μmuL and incubated for another 4 h. The formazan crystals formed by viable cells were then solubilized in DMSO and measured at 490 nm for the absorbance (A) values.

### Soft agar colony formation assay

The anchorage-independent growth of hepatocytes was monitored by the soft agar colony formation assay. In brief, cells (100/well) resuspended in 1.5-ml mixture of 1.2% low-melt agarose and 2×RPMI 1640 (v:v = 1:1) were loaded in triplicate on the top of the solidified bottom agar comprising equal-volume mixture of 0.7% low-melt agarose and RPMI 1640 in 12-well plates. The cells were incubated at 37°C, 5% CO2 for two weeks. The colonies composed of more than 50 Cells were counted.

### In vitro migration and invasion assay

Cells growing in the log phase were treated with trypsin and resuspended as single-cell solution. Then the cells were counted and 1 × 10^5 ^cells in 1 mL of serum-free RPMI 1640 medium were split into the upper chamber of transwell and boyden chamber (8-μmum pore size, BD Biosciences, USA) where the transwell membrane was coated either with (for invasion) or without (for migration) matrigel. Serum-free medium was also added to the lower chamber. The migration assay proceeded in 37°C, 5%CO_2 _tissue culture incubator for 12 h and the invasion assay, 18 h. The non-migrated/invaded cells in the upper chamber were removed using cotton swap, and the migrated/invaded cells fixed in methanol and stained with eosin for migrated cells or Gimsa for invaded cells. The cells trapped in or attached to the reverse side of the porous membrane were photographed through × 200 microscope objective, the numbers of migrated cells in at least 5 random fields counted under phase contrast microscope, and the average calculated.

### In vivo xenograft tumor growth in nude mice

All animal protocols were approved by the Animal Care and Use Committee of Central South University. Nude mice between 4 to 6 weeks were purchased from the Animal Center, Central South University (N = 5). 1 × 10^6 ^cells growing in log phase were resuspended in serum-free RPMI 1640 medium and injected subcutaneously into the 4-6 week-old male BALB/c nu/nu mice. To minimize individual difference, shCtrl cells and shHDGF cells were injected symmetrically to the left-right flank of the same mouse. The mice were maintained in a barrier facility on HEPA-filtered racks. The animals were fed with an autoclaved laboratory rodent diet. All animal studies were conducted in accordance with the principles and procedures outlined in the National Institutes of Health (NIH) Guide for the Care and Use of Animals under assurance number A3873-1. 15 days later, the mice were sacrificed with tumors isolated and their sizes and weights measured. Finally, the HDGF expression was examined again by quantitative real-time PCR in implanted nude mice of shRNA-HDGF and control cell groups. GAPDH gene was used as a normalizing control. The designed paired primers were as follow, HDGF forward, 5' CAGCCAACAAATACCAAGTCT3' reverse: 5' GTTCTCGATCTCCCACAGC 3' GAPDHforward, 5' GAAGGTCGGAGTCAACGG 3' reverse, 5' TGGAAGATGGTGATGGGATT 3'. The PCR reaction was carried out in a volume of 25 μl using SYBR Green Mix(Tiangen Inc,China) on MXP3000 Instrument (Stratagene Laboratory). Experiments were repeated three times.

### Statistical analysis

All data were analyzed by SPSS 13.0 software and presented as mean ± SD. The χ^2 ^test was applied to analyze the relationship between HDGF expression and clinicopathologic characteristics. Survival curves were plotted by the Kaplan-Meier method and compared by the log-rank test. The significance of various variables for survival was analyzed by the Cox proportional hazards model in the multivariate analysis. One-way ANOVA was applied to test the differences between groups for all *in vitro *analyses. ANOVA test was used for the *in vivo *xenograft experiment. A *P *value of less than 0.05 was considered statistically significant.

## Results

### Immunohistochemical analysis of HDGF protein expression in HCC and normal liver tissues

**Figure 1:**
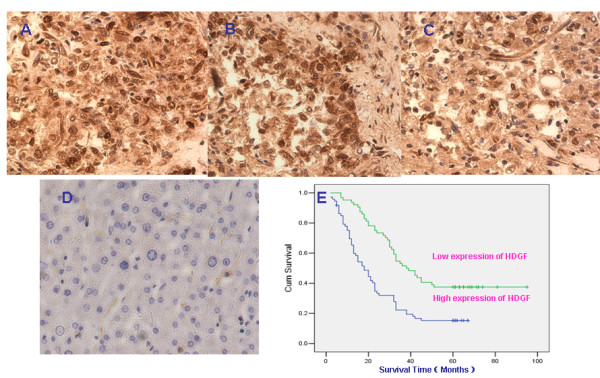
**Detection of HDGF protein in HCC and Kaplan-Meier plots of overall survival duration in patients with HCC**. HDGF protein expression in HCC and normal liver samples. **A and B**. Strong expression of HDGF in HCC samples. **C**. Weak expression of HDGF in HCC samples(original magnification 400×). **D**. Negative expression of HDGF in liver sample; **E**. Kaplan-Meier survival analysis of overall survival duration in 137 HCC patients according to HDGF protein expression. The log-rank test was used to calculate p values.

We measured the expression level and subcellular localization of HDGF protein in 137 archived paraffin-embedded HCC samples and 49 noncancerous samples using immunohistochemical staining expression. Specific HDGF staining was mainly founded in the nuclei and cytoplasm of noncancerous and malignant epithelial cells. We observed that 53.4% (73/137) archival HCC biopsies showed HDGF EI levels 2 for the nuclei. In comparison, the rate of EI levels 2 for HDGF protein expression was 32.7%(16/49) in the nuclei of normal epithelial cells(Figure 1A-D).

### Correlation between HDGF expression and clinicopathologic characteristics in Chinese HCC

We did not find a significant association of HDGF expression in nuclei of tumor cells with age, gender, HBV infection, smoking, drinking, T classification and distant metastasis in patients with HCC (*p *> 0.05). Interestingly, we observed that the nuclear HDGF expression was closely correlated with T classification (*p *< 0.001), N classification (*p *< 0.001) and clinical stage (*p *< 0.001) in patients with HCC(Table 1).

**Table 1:** Correlation between the clinicopathologic characteristics and expression of HDGF protein in liver cancer

Characteristics	n	HDGF (%)		*P*
		High expression	Low expression	
Group				
Cancer tissue	137	73(53.3%)	64 (46.7%)	
Normal tissue	49	16(32.7%)	33 (67.3%)	0.011
Gender				
Male	87	51(58.6%)	36 (41.4%)	
Female	50	22(44%)	28 (56%)	0.112
Age(y)				
≥50	69	36 (52.2%)	33 (47.8%)	
< 50	68	37(54.4%)	31(45.6%)	0.865
Smoking				
Yes	47	24 (51.1%)	23 (48.9%)	
No	90	49 (54.4%)	41 (45.6%)	0.722
Drinking wine				
Yes	48	28(58.3%)	20(41.7%)	
No	89	45(50.6%)	44(49.4%)	0.473
HBV Infection				
Yes	103	54(52.4%)	49(47.6%)	
No	34	19(55.9%)	15(44.1%)	0.843
T classification				
T_1_-T_2_	98	42 (42.9%)	56(57.1%)	
T_3_-T_4_	39	31 (79.5%)	8(20.5%)	0.000
N classification				
N_0_-N_1_	64	22 (34.4%)	42 (65.6%)	
N_2_-N_3_	73	51 (69.9%)	22 (30.1%)	0.000
Distant metastasis				
Yes	33	22 (66.7%)	11 (33.3%)	
No	104	51 (49%)	53 (51%)	0.109
Clinical stage				
I~II	56	11(19.6%)	45 (80.4%)	
III~IV	81	62 (76.5%)	19(23.5%)	0.000

### Survival analysis

To investigate the prognostic value of HDGF for HCC, we assessed the association between HDGF expression and survival duration using Kaplan-Meier analysis with the log-rank test. The log-rank test showed that the survival time of patients with HCC was significantly different between the two groups with HDGF EI level 1 and 2 (*p *< 0.001). In patients with HCC, the high HDGF expression group had shorter survival, whereas the low HDGF expression group had better survival (Figure 1E).

In addition, N classification and clinical stage were also significantly correlated with survival in Kaplan-Meier analysis and log-rank test (for N classification, *p *= 0.028; for clinical stage, *p *= 0.041). To determine whether expression of HDGF is an independent prognostic factor for HCC, we performed multivariate survival analysis of HDGF protein expression and factors including with age, gender, smoking, drinking, HBV infection, T classification, N classification, distant metastasis, or clinical stage in patients with HCC. The results showed that expression of HDGF protein was an independent prognostic factor for HCC (Table 2).

**Table 2:** Summary of univariate and multivariate Cox regression analysis of overall survival duration

Parameter	Univariate analysis			Multivariate analysis		
	
	*P*	HR	95%CI	*P*	HR	95%CI
Age						
≥ 50vs. < 50 years	0.321	1.219	0.825-1.801			
Gender						
Male vs. female	0.310	1.229	0.826-1.829			
Smoking						
Yes vs. No	0.784	1.059	0.701-1.601			
Wine						
Yes vs. No	0.468	0.861	0.574-1.291			
HBV infection						
Yes vs. No	0.866	1.040	0.661-1.634			
T classification						
T_3_-T_42 _vs. T_1_-T	0.390	1.202	0.790-1.828			
N classification						
N2--N3 vs. N0 μ-N1	0.028	1.557	1.048-2.314	0.267	0.722	0.406-1.283
M classification						
M_0 _vs. M_1_	0.170	0.731	0.468-1.143			
Clinical stage						
III-IV vs.I-II	0.041	1.526	1.017-2.290	0.335	1.443	0.684-3.044
HDGF Expression Index(EI)						
II vs.I	0.000	2.316	1.548-3.464	0.000	0.358	0.210-0.610

HR: hazard ratio; CI: confidence interval

### HDGF was highly expressed in HepG2 cells

**Figure 2:**
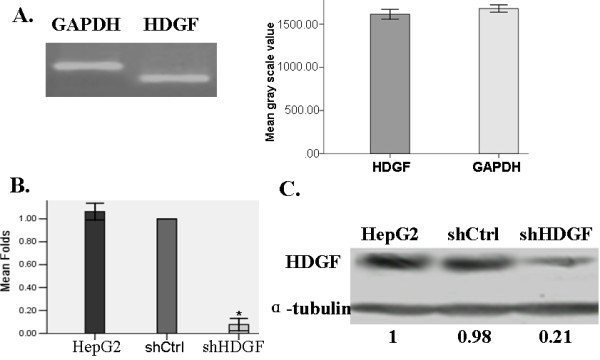
**HDGF was highly expressed in HepG2 cells and this level was successfully knocked down by shRNA**. A. Total RNA was extracted from HepG2 cells and RT-PCR was applied to examine the steady-state mRNA levels of HDGF and GAPDH. **B and C**. Parental HepG2 cells and those stably transfected with either control (shCtrl) or HDGF-specific (shHDGF) shRNA were examined for HDGF expression by quantitative real-time PCR and Western-blot. GAPDH and α-tubulin were used as internal control, respectively.

To examine the biological functions of HDGF, we first measured the expression level of endogenous HDGF in HepG2 cells by RT-PCR. As shown in Figure 2A, when the PCR cycle number was controlled (28 cycles) so that both the HDGF and GAPDH products were in the linear range, HDGF showed an expression level comparable to that of the housekeeping gene GAPDH, suggesting HDGF is highly expressed in HepG2 cells, which also indicates that HepG2 is a good model system for studying the functions of endogenous HDGF by loss-of-function approach.

### Endogenous HDGF was successfully knocked down by shRNA-expressing lentiviral vector

To stably knock down the endogenous expression of HDGF, we applied a lentiviral vector expressing specific shRNA sequence targeting HDGF (shHDGF). As a control, we stably transfected the HepG2 cells with the same lentiviral vector expressing a control shRNA sequence (shCtrl) not targeting any known human genes. By mRNA and protein expression analysis, we found that the shCtrl cells have similar HDGF level as the parental HepG2 cells, which were significantly higher than that in the shHDGF cells (Figure 2B,C).

### HDGF knockdown inhibited the growth of HepG2 cells

**Figure 3:**
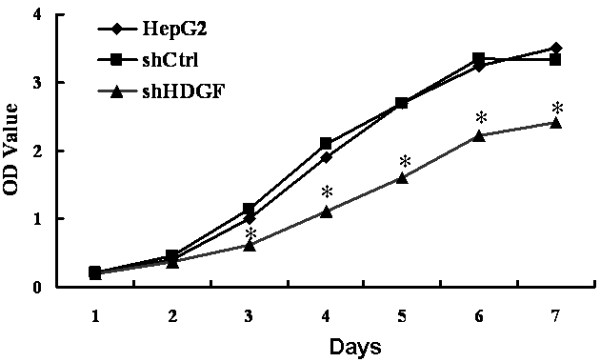
**Down-regulation of HDGF inhibited cell growth**. The cell growth of parental HepG2 cells and their stable derivatives, shCtrl and shHDGF, were examined by MTT assay over a seven-day period. *P < 0.05, as compared to parental HepG2 cells and shCtrl cells.

After successfully knocking down the endogenous expression of HDGF, we first examined its effect on cell growth. As shown in Figure 3, the parental HepG2 cells had a similar growth rate as the shCtrl cells over a seven-day period, while starting from day 3 the growth of shHDGF cells were significantly slower than the former two cells (*P *< 0.05), suggesting HDGF promotes the growth of HepG2 cells.

### HDGF knockdown inhibited cellular transformation

**Figure 4:**
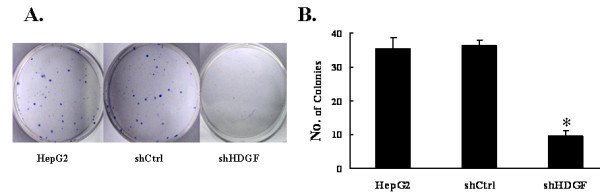
**Down-regulation of HDGF inhibited cell transformation**. The anchorage-independent growth of parental HepG2 cells and their stable derivatives, shCtrl and shHDGF, were examined by soft agar colony formation assay. **A**. Colonies were photographed. B. Bar graph showed the differences of colony formation among the three groups. Data were presented as mean ± SD for three independent experiments. *P < 0.05, as compared to parental HepG2 cells and shCtrl cells.

We next explored the effect of HDGF on cellular transformation. Since anchorage-independent growth is a hallmark for transformed cells, we measured the growth of different cells on soft agar. Both the parental HepG2 cells and the shCtrl cells formed similar number of colonies on soft agar over a two-week period [(35.3 ± 3.5) vs. (36.7 ± 3.5)]. In contrast, knocking down endogenous HDGF dramatically reduced the number of colonies (9.3 ± 2.0)(*p *< 0.05), implying an essential role of HDGF in regulating cellular transformation (Figure 4).

### HDGF knockdown reduced cell migration and invasion

**Figure 5:**
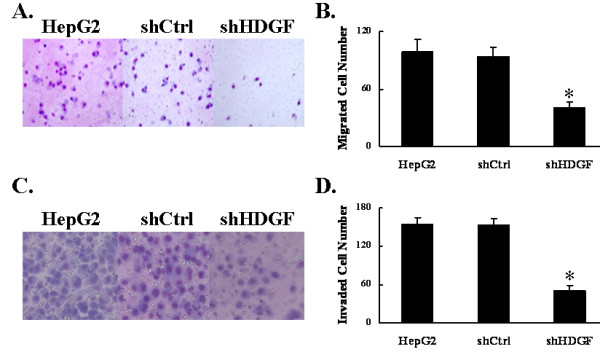
**Down-regulation of HDGF reduced cell migration and invasion**. The migrating (A and B) and invading (C and D) capabilities of parental HepG2 cells and their stable derivatives, shCtrl and shHDGF, were examined by transwell and boyden chamber assay. **A and C**. Migrated or invaded cells were photographed under the microscope (200×). **B and D**. Quantifications of migration and invasion were presented as mean ± SD for three independent experiments. *P < 0.05, as compared to parental HepG2 cells and shCtrl cells.

Cell migration and invasion are integral steps for the process of tumor development and metastasis. When testing the abilities of HepG2 cells to migrate/invade through 8-μmum pores on the polycarbonate membrane either without or with pre-coated matrigel, we found the knocking down endogenous HDGF significantly reduced the potentials of HepG2 cells to both migrate and invade (*p *< 0.05), as compared to the parental or shCtrl cells (Figure 5).

### HDGF contributes to in vivo xenograft tumor growth

**Figure 6:**
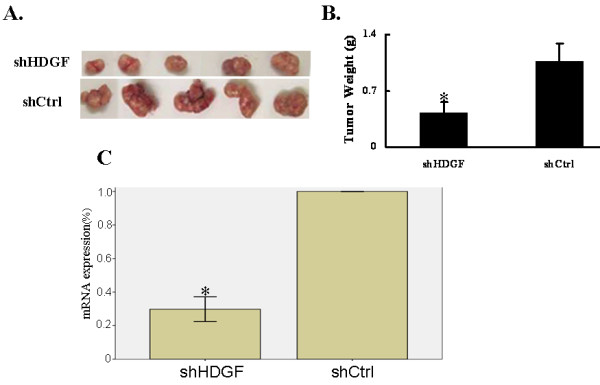
**Down-regulation of HDGF inhibited *in vivo *xenograft tumor growth**. A. shCtrl and shHDGF cells were injected subcutaneously into nude mice (N = 5 for each group) and the tumors were isolated two weeks later. B. Tumor weights were quantified from 5 mice and presented as mean ± SD. *P < 0.05, as compared to shCtrl cells.

In addition to examining the biological functions of HDGF *in vitro*, we also assessed the *in vivo *function of HDGF using a xenograft transplantation model. By subcutaneously transplanting the shCtrl or shHDGF cells into nude mice, we monitored the tumor growth over a 15-day period. As shown in Figure 6, by measuring the tumor weights, we found that shHDGF cells gave rise to significantly smaller tumors than shCtrl cells (*p *< 0.05). Real-time qPCR showed that HDGF expression was obviously reduced in implanted nude mice of shHDGF cell groups compared with control cell groups(Figure 6C).

## Discussion

Hepatocellular carcinoma (HCC) is one of the most common malignancies with extreme poor prognosis. It was reported that in patients with symptomatic HCC, the five-year survival rate is less than 5% [1]. On the molecular level, a number of epigenetic and genetic events have been associated with the development of HCC, including inactivation of tumor suppressor p53 and epigenetic targeting of cancer-related genes such as HIA-2, CDKN2A, p16-INK4a, E-cadherin and T-cadherin, activation of JNK1, ErbB-2, Wnt signaling and multiple receptor tyrosine kinases[24-31]. All these genetic and epigenetic alterations are not unique for HCC, but also present in many other human malignancies. Among the various changes, the up-regulation of hepatoma-derived growth factor (HDGF) is gaining increasing attentions. HDGF was first cloned from the conditioned medium of hepatoma cell line HuH-7 and was found to be an acidic, heat-labile heparin-binding protein with mitogenic activity for fibroblasts [4]. Although initially identified as a secreted trophic factor, recent evidence suggested that the nuclear targeting of HDGF is essential for its mitogenic activity [32,33]. Consistent with its function as a mitogen, HDGF is found to be up-regulated in multiple cancers and its expression level negatively correlates with the prognosis of cancer patients including gastric cancer, HCC in Japan populations, non-small-cell lung cancer, esophageal carcinoma, and pancreatic cancer [13-19], implying its importance during cancer development. However, except for its mitogenic activity, the expression role of HDGF in Chinese population and the molecular understandings on HDGF actions are quite limited, with most acquired from gain-of-function studies [8,34,35]. In the present study, we utilize immunohistochemistry to evaluate protein expression of HDGF in HCC and further analyze its protein expression in clinicopathologically characterized 137 HCC cases.

HDGF protein was highly expressed in HCC tissues compared with normal liver tissues. Subsequently, we analyzed the correlation of HDGF expression with clinicopathologic features in HCC. Inconsistent with Yoshida's report [15], our results indicated that significantly increased nuclear protein expression of HDGF closely associated with T classification (*p *< 0.001), N classification (*p *< 0.001), clinical stage (*p *< 0.001) in patients with HCC, which hinted that HDGF as a growth factor might play an important role in HCC genesis and progression rather than distant metastasis(*p *= 0.109). The discrepancy between our data and Yoshida's data would be most likely due to the different samples and evaluation criterion used for immunohistochemistry. Furthermore, we also found that the level of nuclear HDGF protein expression was markedly correlated with overall survival. In multivariate analyses, a high level of expression of HDGF protein was associated with a poor prognosis for HCC. This result indicated that HDGF is a potential unfavorable prognostic factor for Chinese HCC patients. Similar to our result, Yoshida et al also reported that HCC patients from Japan with a positive HDGF index had significantly poorer disease-free and overall survivals compared with patients with a negative index [15].

Further, we analyzed the function of HDGF in HepG2 cells. By transfecting the cells with shRNA-expressing lentiviral vector followed by selection with Blasticidin, we successfully established stable cells expressing either control or HDGF-specific shRNA, with the latter showing dramatically reduced HDGF level as compared to the former. The subsequent functional studies demonstrated that knocking down the endogenous expression of HDGF led to significant reduced *in vitro *cell growth, transformation, migration, invasion, as well as *in vivo *xenograft tumor formation. Compared with the previous gain-of-function studies, this loss-of-function study offers more insights on the functions of endogenous HDGF, minimizing the confounding factors that might be introduced by overexpressed HDGF at a physiologically irrelevant high level. Our study also revealed a novel function of HDGF in HCC, that is, to promote the cell migration and invasion, suggesting its potential involvement in cancer metastasis. This is consistent with the finding by Zhang *et al*. that down-regulation of HDGF inhibits the invasion of non-small cell lung cancer cells [36], which further indicates that the biological functions of HDGF are not unique to a specific cancer, but common to multiple cancers. The observations that HDGF regulates multiple cellular processes such as cell growth, cell transformation, migration, invasion, and is a prognostic factor for multiple cancers implies its importance as a therapeutic target for treating multiple human cancers, including HCC. This is further supported by the recent work of Ren *et al*. that provided the experimental evidence of targeting HDGF as a strategy for treating lung cancer [37].

## Conclusion

In summary, we found that elevated expression of nuclear protein expression of HDGF was not only showed in HCC tissue compared with noncancerous liver tissues, but also closely associated with T classification, N classification, clinical stage in patients with HCC. Furthermore, we also found that the level of nuclear HDGF protein expression was significantly correlated with overall survival. In multivariate analyses, a high level expression of nuclear HDGF protein was associated with a poor prognosis for HCC. We applied shRNA-mediated gene knockdown approach and examined the biological processes regulated by HDGF in HepG2 cells. We demonstrated the functional importance of HDGF in cell growth, transformation, migration and invasion, providing the directions for future studies on molecular mechanisms of HDGF.

## Abbreviations

HCC: Hepatocellular carcinoma; HDGF: Hepatoma-derived growth factor; UICC: the International Union Against Cancer; qPCR: Quantitative Real Time-Polymerase Chain Reaction; RT-PCR: Reverse Transcription Polymerase Chain Reaction

## Competing interests

The authors declare that they have no competing interests.

## Authors' contributions

All authors have read and approved the final manuscript. JRH and WYF set up the protocols, YYZ and NXZ contributed in the experimental procedures and in the interpretation of the data, JRH and WYF supervised all the work and wrote the paper.
